# Metabolic and gut microbiome changes following GLP-1 or dual GLP-1/GLP-2 receptor agonist treatment in diet-induced obese mice

**DOI:** 10.1038/s41598-019-52103-x

**Published:** 2019-10-30

**Authors:** Mette Simone Aae Madsen, Jacob Bak Holm, Albert Pallejà, Pernille Wismann, Katrine Fabricius, Kristoffer Rigbolt, Martin Mikkelsen, Morten Sommer, Jacob Jelsing, Henrik Bjørn Nielsen, Niels Vrang, Henrik H. Hansen

**Affiliations:** 1 Gubra, Hørsholm, Denmark; 2 Novo Nordisk Foundation Center for Biosustainability, Technical University of Denmark, Kgs. Lyngby, Denmark; 3 Clinical Microbiomics, Ole Maaløes Vej 3, Copenhagen, Denmark

**Keywords:** Pharmacokinetics, Metagenomics

## Abstract

Enteroendocrine L-cell derived peptide hormones, notably glucagon-like peptide-1 (GLP-1) and glucagon-like peptide-2 (GLP-2), have become important targets in the treatment of type 2 diabetes, obesity and intestinal diseases. As gut microbial imbalances and maladaptive host responses have been implicated in the pathology of obesity and diabetes, this study aimed to determine the effects of pharmacologically stimulated GLP-1 and GLP-2 receptor function on the gut microbiome composition in diet-induced obese (DIO) mice. DIO mice received treatment with a selective GLP-1 receptor agonist (liraglutide, 0.2 mg/kg, BID) or dual GLP-1/GLP-2 receptor agonist (GUB09–145, 0.04 mg/kg, BID) for 4 weeks. Both compounds suppressed caloric intake, promoted a marked weight loss, improved glucose tolerance and reduced plasma cholesterol levels. 16S rDNA sequencing and deep-sequencing shotgun metagenomics was applied for comprehensive within-subject profiling of changes in gut microbiome signatures. Compared to baseline, DIO mice assumed phylogenetically similar gut bacterial compositional changes following liraglutide and GUB09-145 treatment, characterized by discrete shifts in low-abundant species and related bacterial metabolic pathways. The microbiome alterations may potentially associate to the converging biological actions of GLP-1 and GLP-2 receptor signaling on caloric intake, glucose metabolism and lipid handling.

## Introduction

Obesity has become a worldwide epidemic and is an important driver for the increasing prevalence of type 2 diabetes and other co-morbidities^1,2^. Obesity is a complex condition involving multifactorial regulatory mechanisms, including metabolic, endocrine, and neuroadaptive responses to chronic overconsumption of energy-dense high-fat/carbohydrate diets^3,4^. An increasing number of studies have also implicated gut microbial imbalances (‘gut dysbiosis’) in the development of obesity. Although gut dysbiosis has yet to be causally linked to obesity in humans, accumulating evidence demonstrate distinct taxonomic shifts in gut microbial communities of obese individuals compared to lean subjects^5–8^. Diet-induced obese (DIO) mouse models have been instrumental in implicating gut microbiota in obesity. A range of studies have indicated the presence of specialized obesity-associated gut microbiota which may favor production of microbial fermentation products and metabolites increasing efficiency of energy harvest from ingested nutrients as well as modulating gut motility, permeability and immune function^9–13^. The clearest demonstration of aberrant gut microbiota-host interaction in obesity comes from studies in germ-free mice, demonstrating that the obese phenotype is transmissible from DIO mouse donors to lean recipients and vice versa^9,14,15^.

There is an increasing focus on the potential contributory mechanistic role of the gut microbiota in the metabolic effects of glucoregulatory and weight loss promoting drugs, including glucagon-like peptide-1 (GLP-1) receptor agonists, which have become well-established therapies in type 2 diabetes and obesity^16,17^. In addition to the glucoregulatory and central appetite-suppressive effects of GLP-1 receptor agonists, this drug class also exhibit gastrointestinal effects by inhibiting gastric emptying as well as having intestinotrophic and gut immunomodulatory properties^18–22^. A closely related and co-secreted L-cell peptide hormone, glucagon-like peptide-2 (GLP-2), is a potent inducer of gut epithelial proliferation, stimulates nutrient absorption, improves mucosal integrity and reduces gut permeability^23–25^. Interestingly, GLP-1/GLP-2 co-agonists have recently been reported to have greater glycemic and intestinotrophic effects compared to the GLP-1 receptor agonist, liraglutide^26^. Although it is presently unresolved whether the gut microbiota plays a role in the effects of these peptide hormones, it has been speculated that host-gut microbiota crosstalk, facilitated by the production and signaling of bacterial fermentation end-products of dietary fibers (short-chain fatty acids), influences intestinal chemo-sensing and modulates the release and activity of gut peptides^27–30^.

Knowledge in this area is still sparse as structural modulation of the gut microbiome has only been reported at a relative low level of resolution (phylum/genus level) following treatment with agonists selective for the GLP-1^31–34^ and GLP-2^25,35^ receptors. In addition, most current approaches for analyzing gut microbiome data rely on comparisons to reference genomes^36^. However, the microbial diversity extends far beyond what is covered by reference databases and segregation of gut communities into specific bacterial species therefore remains a largely unsolved problem.

Recently, methods have been developed for more comprehensive profiling of the diversity within complex gut microbiota samples, including deep-sequencing shotgun metagenomics^37–39^. Here, we applied these methods to compare the gut microbiome composition in DIO mice before and after long-term treatment with liraglutide or a dual GLP-1/GLP-2 receptor agonist, GUB09-145^26^.

## Materials and Methods

### Animals

Four weeks-old male C57BL/6 mice (Janvier Labs, Saint Berthevin, Cedex, France) were housed in a controlled environment (12 h light/dark cycle, lights on/off at 3AM/3PM; 22 ± 1 °C; 50 ± 10% relative humidity). Each animal was identified by an implantable microchip (PetID Microchip, E-vet, Haderslev, Denmark). Mice were fed either chow (Altromin #1324, energy density 2.85 kcal/g, Brogaarden, Lynge, Denmark) or a high-fat diet (60% kcal fat, #D12492, energy density 5.21 kcal/g, Ssniff, Soest, Germany) for 35 weeks prior to drug treatment. Diets and domestic quality tap water were available *ad libitum*. All animal experiments were approved by the Danish Committee for Animal Research using internationally accepted principles for the use of laboratory animals (license no. 2013-15-2934-00784).

### Drug treatment

Liraglutide was from Hoersholm Pharmacy (Hoersholm, Denmark). GUB09-145 was synthesized at Gubra by automated solid-phase peptide synthesis^26^. Both compounds were freshly dissolved in vehicle (PBS with 0.1% BSA). All animals were single-housed two weeks prior to treatment and throughout the remainder of the study period. DIO mice were randomized to treatment according to body weight (n = 15 per group) and dosed subcutaneously (5 ml/kg) for a total of 28 days with vehicle (QD), liraglutide (0.2 mg/kg, BID) or GUB09-145 (0.04 mg/kg, BID). The liraglutide and GUB09-145 doses administered in the present study are within dose ranges previously reported to promote a robust weight loss in DIO mice^26,40–42^. Chow-fed control mice (n = 10) received a subcutaneous injection with vehicle (QD). Body weight and food intake were measured daily during the treatment period.

### Oral glucose tolerance test

An oral glucose tolerance test (OGTT) was performed on treatment day 27. Animals were fasted 4 h prior to the OGTT. Vehicle or compounds were administered 60 min prior to the OGTT. At time 0, a bolus of glucose (2 g/kg, 10 ml/kg, Fresenius Kabi, Uppsala, Sweden) was administered by oral gavage. Tail vein blood samples were collected in 10 µl heparinized capillary tubes at times −60, 0, 15, 30, 60, 120 and 240 min., and immediately suspended in glucose/lactate solution buffer (0.5 mL, EKF-diagnostics, Cardiff, UK). Blood glucose concentrations were measured using a BIOSEN C-Line glucose meter (EKF-diagnostics, Barleben, Germany) according to the manufacturer’s instructions.

### Plasma biochemistry

Blood glucose levels were measured in tail blood samples from 4 h fasted mice on treatment day 28. Animals were terminated by cardiac puncture under isoflurane anesthesia. Cardiac blood samples were collected in heparinized tubes and centrifugated (1,500 × g, 10 min, 4 °C). Terminal plasma samples were assayed for insulin, total triglyceride (TG) and total cholesterol (TC) concentrations. Insulin was measured in duplicates using an AlphaLisa kit (Perkin Elmer, Skovlunde, Denmark), according to the manufacturer’s instructions. TG and TC were determined on a Cobas 6000 autoanalyzer (Roche, Basel, Switzerland), according to the manufacturer’s instructions. Assay data (glucose area-under-the curve (AUC), TG, TC, fasted glucose, fasted insulin) were evaluated by a 1-way ANOVA or a two-way ANOVA (body weight, food intake, OGTT glucose), in both cases the p-values were corrected for multiple testing using the Bonferroni method.

### DNA extraction

Fresh fecal samples were collected on treatment day −1 and 27, respectively. Samples were taken directly from the anus or from a clean empty cage if immediate defecation did not occur. The samples were placed on dry ice until sampling was complete, then stored at −80 °C until further processing. Bacterial DNA was extracted using the 96-well NucleoSpin Soil DNA Isolation Kit (#740787, Macherey-Nagel, Düren, Germany). Average bacterial DNA amount in mouse fecal samples was 1.00 µg (range 0.02–6.9 µg). A positive mock sample (bacterial culture, 0.08 µg DNA) was included to control for DNA extraction efficiency.

### 16S rRNA amplicon sequencing

All paired samples were evaluated by 16S rDNA amplicon sequencing (chow, n = 10; DIO-vehicle, n = 15; DIO-liraglutide, n = 15; DIO-GUB09-145, n = 15). 16S rDNA amplicon libraries were prepared targeting the V3 and V4 hypervariable regions of the ribosomal DNA. The purified genomic DNA served as the template and was amplified by PCR (98 °C for 30 sec, 25 × (98 °C for 10 s, 55 °C for 20 s, 72 °C for 20 s), 72 °C for 5 min) with the forward primer S-D-Bact-0341-b-S-17 (5′-TCGTCGGCAGCGTCAGATGTGTATAAGAGACAGCCTACGGGNGGCW-GCAG-3′) and reverse primer S-D-Bact-0785-a-A-21 (5′-GTCTCGTGGGCTCGG-AGATGTGTATAAGAGACAGGACTACHVGGGTATCTAATCC-3′) with Illumina adapters attached. Indexes were added in a subsequent PCR (98 °C for 30 sec, 8 × (98 °C for 10 s, 55 °C for 20 s, 72 °C for 20 s), 72 °C for 5 min) using the Illumina Nextera Index Kit V2 and the attachments were verified by running the products on an agarose gel. Products from the nested PCR were pooled and the resulting library purified with magnetic beads. The DNA concentration of the pooled libraries was measured fluorometrically. The final 16S rDNA amplicon libraries were sequenced on an Illumina MiSeq using the MiSeq Reagent Kit V3 (Illumina) for 2× 300 bp paired-end sequencing.

### Microbiome diversity and richness analysis

The 16S rDNA amplicons were analysed on rarefied data with 13,730 reads per sample. A 64-bit version of USEARCH and mothur were used in combination with bioinformatic tools developed and reported previously^43,44^. After tag identification and trimming, all sequences from all samples were pooled and paired-end reads merged. Ambiguous and error prone sequences were discarded, and remaining sequences were clustered into operational taxonomic units (OTUs) with 97% sequence similarity using USEARCH^43^. Chimeric OTUs were discarded based on comparison with the Ribosomal Database Project classifier training set v9 using UCHIME^45,46^. Taxonomic assignment of the OTUs was done using the database from the Ribosomal Database Project^47^. Species richness, Shannon diversity index and Bray-Curtis dissimilarities were computed and principal coordinate analyses (PCoA) were conducted with R^48,49^. Differences in species richness and Shannon diversity between paired samples at the two timepoints were assessed using a two-sided Wilcoxon signed-rank test. Compositional differences between study groups were measured using Bray-Curtis dissimilarity, which was calculated based on the OTU abundances and projected on the first two dimensions of a PCoA plot^49^. Both sample timepoints were included in the distance matrices, though the resulting PCoA plots were split into two time points for illustrative purposes. Comparing compositional similarity was evaluated using PERMANOVA test (Permutational Multivariate Analysis of Variance, Adonis test from the R-package Vegan) and Bray-Curtis dissimilarity. All p-values were corrected for multiple testing using Benjamini Hochberg False Discovery Rate (FDR) and were considered statistically significant when p < 0.05.

### Whole-Genome Shotgun sequencing and data analysis

A subset of the paired samples was further evaluated by whole-genome shotgun sequencing (chow, n = 7; DIO-vehicle, n = 5; DIO-liraglutide, n = 10; DIO-GUB09-145, n = 11). Genomic DNA was randomly cut into fragments of approximately 350 bp by restriction enzyme. NEBNext UltraTM Library Prep Kit for Illumina (New England Biolabs, Ipswich, MA) was used for library construction. The prepared DNA libraries were evaluated using Qubit 2.0 fluorometer quantitation and Agilent 2100 Bioanalyzer for assessment of fragment size distribution. Quantitative real-time PCR was used to detect the concentration of the final library before sequencing. Whole-genome sequencing (WGS) was performed using 2× 150 bp paired-end sequencing on an Illumina HiSeq platform. WGS output was quality trimmed using Trimmomatic removing the first 10 bp and cutting reads at 3′-end with a sliding window of 4 bp and minimum mean phred score at 15 to generate high-quality reads^50^. Reads shorter than 60 bp or mapping to the mouse reference genome were discarded using Bowtie2^51^. *De novo* assembly was performed for each sample using Megahit and contigs at least 500 bp long were used for gene prediction using Prodigal^52,53^. Gene predictions from all samples were combined and metagenomic species (MGS) were created based on a mice gene catalog and by binning genes that were highly co-abundant across samples^37,54^. Taxonomical annotation was performed using BLASTn against reference sequences in the NCBI RefSeq database (version from 120417)^55^. Genes were annotated to species level using 95% identity and 80% coverage; to genus, family, order and class level using 80% identity and 80% coverage, and to phylum level using 65% identity and 50% coverage. Furthermore, a species was assigned a taxonomy to a given level if more than a given percentage of genes were consistently annotated to a specific taxon. For species, this was 80%, for genus 75%, family and order 60%, class 50%, and for phylum 40% of the genes that were consistently annotated to the respective taxonomical level. An MGS counts table was created based on the total gene counts for each MGS. The MGS counts table was downsampled (rarefied) and normalized according to effective gene length (accounting for read length) and then normalized to sum to 100%, resulting in relative abundance estimates of each MGS.

### Metagenomic species clustering and statistics

For species present in 10% or more samples (n = 609) a linear mixed model (LMM) regression was performed to test for abundance changed within each DIO mouse treatment group using the lme4 package in the R environment^56^. Relative abundances were log10-transformed and regressed against the interaction between treatment and sampling time (baseline, termination) and the individual mouse was modelled as a random effect. A post-hoc Tukey test was used to determine which groups differed significantly. For graphical clarity, abundance profiles of changing species were clustered using Partitioning Around Medoids (PAM)^57^ (k = 4, was chosen manually). Correlations between significant differential abundant species and metabolic measures (% body weight loss, total food intake, triglycerides, cholesterol, fasting glucose and AUC glucose) were calculated using a correlation test (Spearman) and corrected for multiple testing (Benjamini Hochberg).

### Functional analysis

Functional annotation was obtained by mapping genes to the eggNOG orthologous database using the Emapper software^58^ in HMMER mode (e-value < 0.001). Functional annotation to the KEGG database was performed using MOCAT2^59,60^. Only, KEGG modules found in >10% of the samples were used for statistical analysis. Abundance statistics for functional annotation was done as described for species abundances.

## Results

### Liraglutide and GUB09-145 improve metabolic parameters in DIO mice

**Figure 1:**
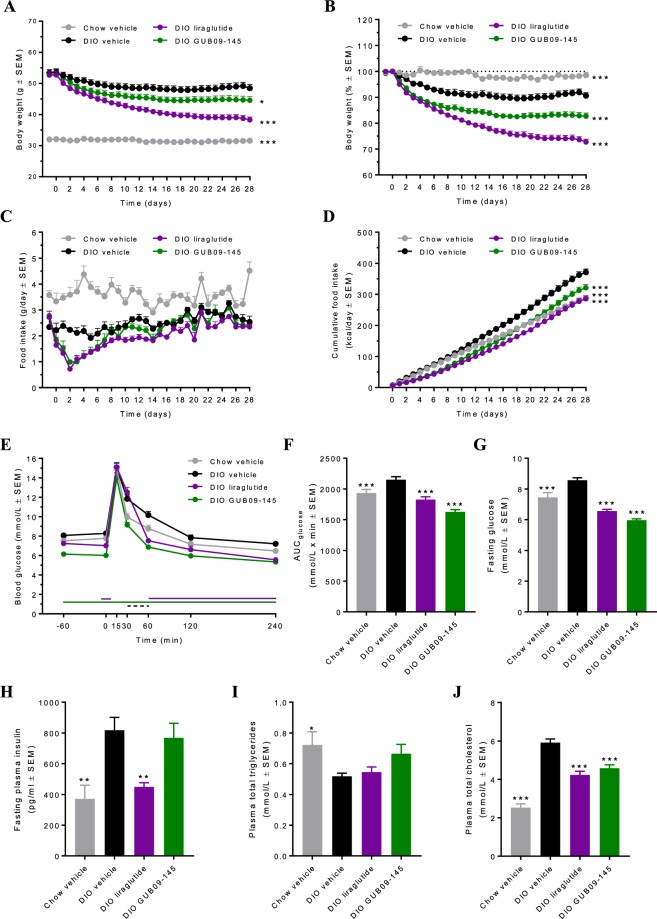
Liraglutide and GUB09-145 improve metabolic parameters in DIO mice. (**A**) Absolute body weight (g), (**B**) Body weight gain (%) relative to treatment start; (**C**) Daily food intake (g); (**D**) Cumulative energy intake (kcal/day); (**E**) Oral glucose tolerance test (OGTT) on treatment day 27, (**F**) Glucose area-under the-curve (glucose AUC_0-240 min_); (**G**) Fasting blood glucose concentrations (mmol/L) on treatment day 14; (**H**) Fasting plasma insulin levels (pg/ml) on treatment day 28; (**I**) Plasma total triglycerides (TG, mmol/L) on treatment day 28; (**J**) Plasma total cholesterol (TC, mmol/L) on treatment day 28. *p < 0.05, **p < 0.01, ***p < 0.001 compared to DIO vehicle controls.

Following 35 weeks of feeding, DIO mice showed significantly increased body weight (53.0 ± 0.6 g, n = 45) compared to chow controls (32.1 ± 0.2 g, n = 10, p < 0.001, Fig. 1A,B). Daily food intake curves are indicated in Fig. 1C. Cumulated energy intake was significantly higher in vehicle-dosed DIO mice (n = 15) compared to chow-fed controls (Fig. 1D, p < 0.001). In addition, vehicle-dosed DIO mice showed mildly impaired oral glucose tolerance at treatment day 27 compared to vehicle-dosed chow controls (Fig. 1E,F). Compared to chow controls, vehicle-dosed DIO mice also displayed significantly increased fasting glucose measured on day 14 (p < 0.001, Fig. 1G) and terminal insulin levels (p < 0.01, Fig. 1H), lowered plasma TG (p < 0.05, Fig. 1I), and elevated TC (p < 0.001, Fig. 1J). A significant weight loss was observed in DIO mice during four weeks of treatment with liraglutide (0.2 mg/kg, SC, BID, n = 15) and GUB09-145 (0.04 mg/kg, SC, BID, n = 15) (p < 0.01, Fig. 1B). Compared to baseline (treatment day 0), terminal weight loss amounted to 27.3 ± 1.1% (14.4 ± 0.7 g) and 17.3 ± 1.2% (9.3 ± 0.6 g) in liraglutide and GUB09-145 treated DIO mice, respectively (Fig. 1A,B). Both compounds significantly reduced high-fat diet intake within the first days of dosing whereafter food intake was gradually normalized (Fig. 1C). Liraglutide and GUB09-145 improved glucose tolerance, with GUB09-145 being slightly more effective (Fig. 1E,F). Compared to vehicle controls, both compounds also improved fasting blood glucose levels (p < 0.001, Fig. 1G) and hypercholesterolemia (p < 0.001, Fig. 1J). Only liraglutide improved hyperinsulinemia in the DIO mice (p < 0.01, Fig. 1H). Compared to chow-fed mice, plasma triglyceride levels were slightly reduced in DIO mice (p < 0.05) and were unaffected by drug treatment (Fig. 1I).

### Gut microbiome structural changes determined by 16S rDNA amplicon sequencing

**Figure 2:**
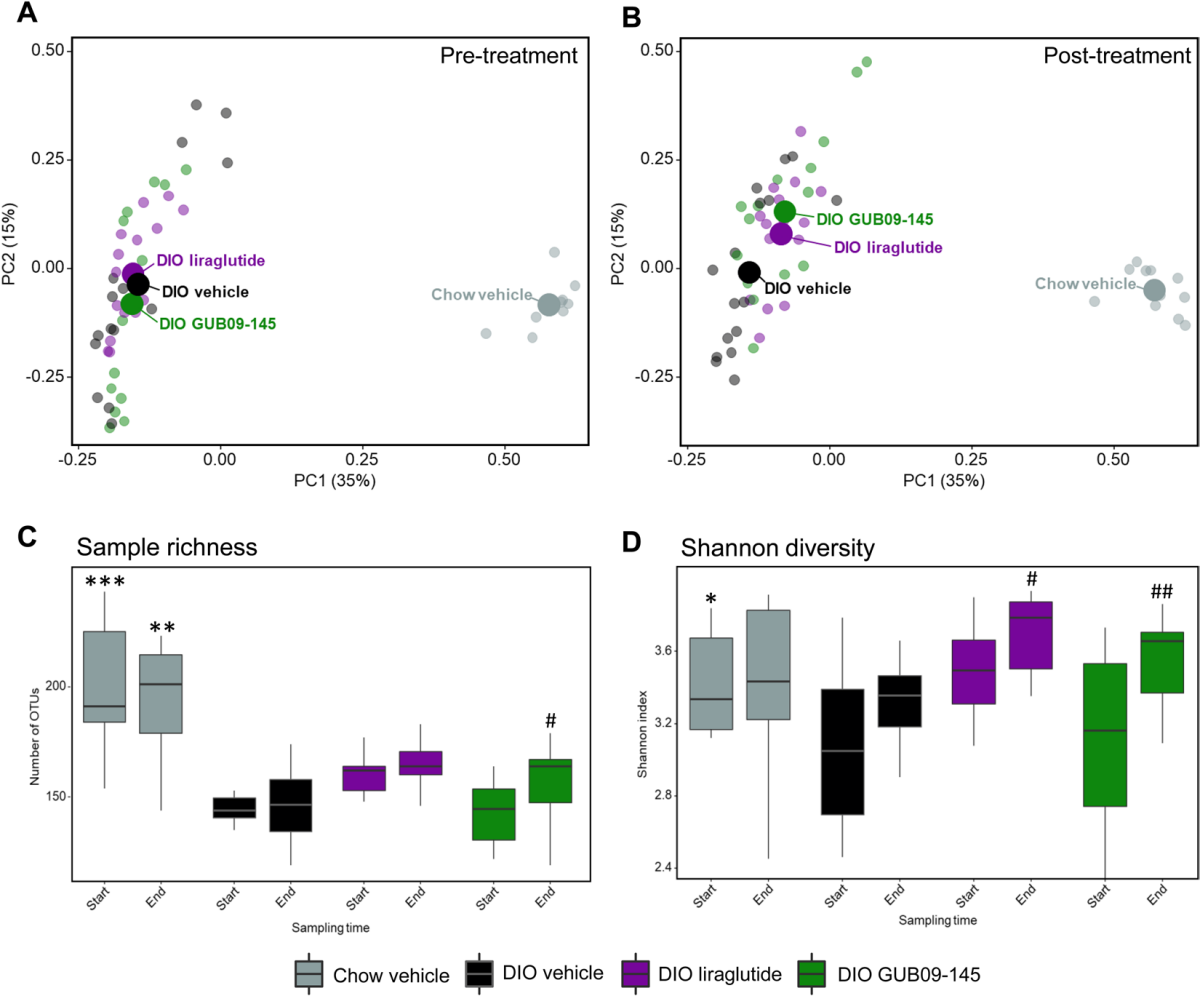
16S rDNA sequencing. Principal Coordinate Analysis (PCoA) based on Bray-Curtis dissimilarity between all samples (panels A,B). PCoA plots show pre- and post-treatment gut microbiome signatures in all individual mice, group means are indicated by a large point. Microbial diversity analysis at genus level, including sample (OTU) richness (**C**) and Shannon alpha diversity (**D**). *p < 0.05, **p < 0.01, ***p < 0.001 vs. corresponding sampling time in DIO vehicle mice; ^#^p < 0.05, ^##^p < 0.01 vs. corresponding baseline.

Baseline and terminal gut bacterial signatures were homogenous within each individual group (Fig. 2A,B). The PCoA plots demonstrated substantial compositional differences (beta-diversity) between chow-fed and DIO mice (r^2^ = 0.49, p = 0.001) which were largely unaffected by vehicle dosing (chow/DIO: r^2^ = 0.06/0.04, p = 0.21/0.25; Fig. 2A,B). In contrast, drug treatment promoted relatively discrete changes in the microbiome signature in DIO mice (liraglutide/GUB09-145: r^2^ = 0.14/0.15, p = 0.001/0.001). In addition, microbial alpha-diversity analyses were performed based on 16S sequencing data. Compared to chow-fed controls, DIO mouse controls showed significantly reduced gut bacterial richness (number of observed OTUs, baseline p < 0.001; termination p = 0.001, Fig. 2C) and Shannon diversity index (which accounts for both species richness and evenness in abundance, baseline p = 0.02; termination p = 0.72, Fig. 2D).

**Figure 3:**
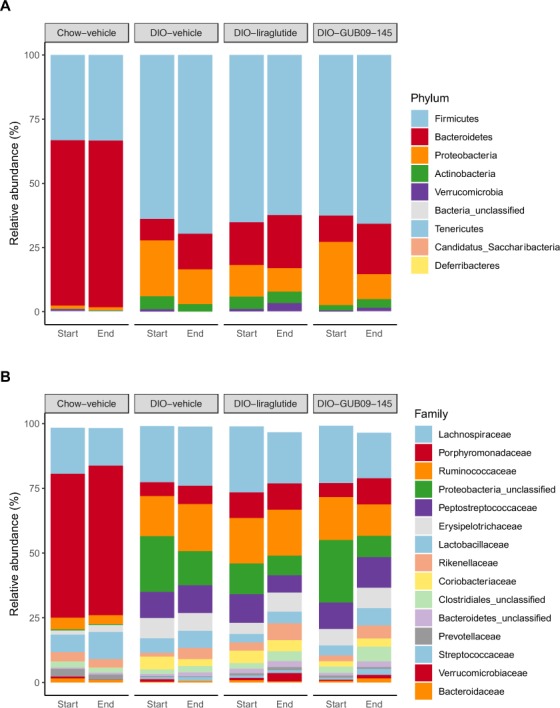
Taxonomic summary at phylum and family level. Mean values per group are illustrated of the nine detected phyla (panel A) and the 15 most abundant families (panel B) across all samples. The phyla and families are sorted with the highest abundance across all samples in the top. For panel B, note that bar height for liraglutide and GUB09-145 (endpoint data) is slightly lower compared to baseline, reflecting that terminal samples showed a relatively higher proportion of reads not mapping to the top-15 most abundant families.

At baseline, all DIO groups showed similar lowered level of bacterial richness and diversity. Dominant phyla in chow-fed controls were *Bacteroidetes*, *Firmicutes*, *Proteobacteria*, and *Actinobacteria* (Fig. 3A). The reduction in bacterial diversity in DIO mice was mainly attributed to enrichment of *Firmicutes* and *Proteobacteria* with a parallel reduction in the abundancy of *Bacteroides* (Fig. 3B). At the bacterial family level, the gut microbiome changes in DIO mice were associated with significantly increased relative abundance of members of the *Firmicutes* phylum (*Peptostreptococcaceae*, p < 0.001), Proteobacteria (*Proteobacteria_unclassified*, p = 0.013), and significant abundance reductions of members belonging to the *Bacteroidetes* phylum driven by a substantial loss of *Porphyromonadaceae* (p < 0.001) and *Prevotellaceae* (p < 0.001) (Fig. 3B).

Compared to baseline, liraglutide and GUB09-145 treatment promoted largely similar directional shifts in DIO mouse gut microbiome signatures. Nevertheless, the microbiome signatures remained highly different from the corresponding profile in chow-fed controls (Fig. 2A,B). Gut microbial (OTU) richness was significantly increased following GUB09-145 treatment (p = 0.03) and tended to be higher after liraglutide treatment although this effect did not attain statistical significance (Fig. 2C). In contrast, vehicle-dosed DIO mice remained at lower gut microbial richness (p = 0.68, Fig. 2C). Shannon diversity changed only in compound-treated DIO mice (vehicle, p = 0.11; liraglutide, p = 0.02; GUB09-145, p = 0.009, Fig. 2D). In all DIO mice, gut microbiome signatures were unchanged at the family level (p_adj_ > 0.05) following treatment with vehicle, liraglutide or GUB09-145 as compared to baseline (Fig. 3B).

### Gut microbiome structural changes determined by deep sequencing shotgun metagenomics

**Figure 4:**
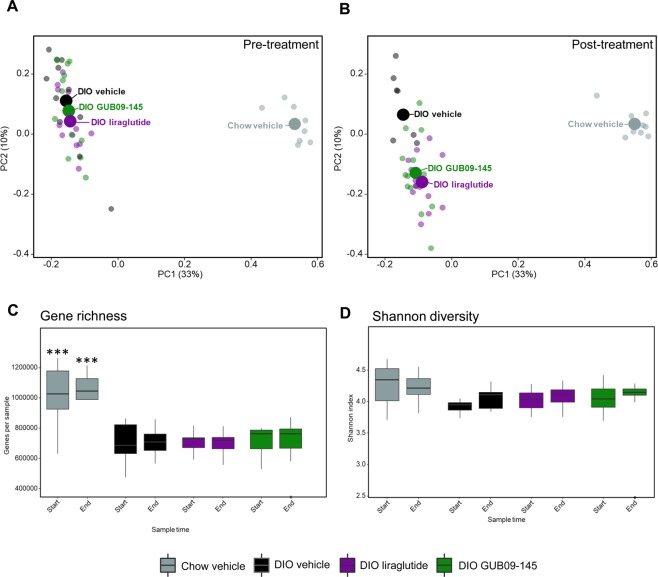
Shotgun metagenomics. Principal Coordinate Analysis (PCoA) based on Bray-Curtis dissimilarity between all samples calculated using species relative abundances (panels A,B). PCoA plots demonstrate pre- and post-treatment gut microbiome signatures in all individual mice, group means are indicated by a large point. Microbial diversity analysis at species level, including gene richness (**C**) and Shannon alpha diversity (**D**). ***p < 0.001 vs. corresponding sampling time in DIO vehicle mice.

Consistent with the finding of reduced bacterial richness and diversity in DIO mice, gene richness was lower in all DIO mouse groups (average 530,000–560,000 genes) compared to chow-fed mice (average 630,000 genes). The shotgun analysis yielded highly similar overall differences in microbiome signatures in the study groups as also identified by 16S sequencing (Figs 2A,B and 4A,B), further supporting the observation that liraglutide and GUB09-145 treatment resulted in larger microbiome compositional changes compared to vehicle-dosed DIO mice. Baseline gene richness measured at the species level was significantly lower in DIO mice compared to chow-fed controls (p < 0.001) and remained lower following vehicle dosing (p < 0.001, Fig. 4C). In contrast, Shannon diversity was similar in chow-fed and DIO mice (Fig. 4D). Compared to baseline, gene richness and diversity did not differ significantly in liraglutide and GUB09-145 treated DIO mice (Fig. 4C,D).

**Figure 5:**
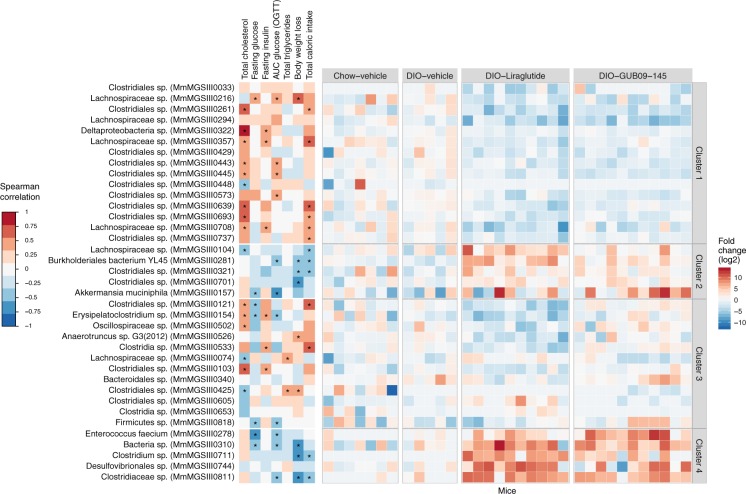
Correlation of metagenomic species abundance to changes in metabolic parameters. Overview of metagenomic species significantly changing in abundance over the course of liraglutide and GUB09-145 treatment. Hierarchical clustering analysis yielded four individual species categories: (*Cluster 1*) species with reduced abundance after liraglutide or GUB09-145 treatment; (*Cluster 2*) species with increased abundance primarily after liraglutide treatment; (*Cluster 3*) species with abundance change specific to one drug treatment group only; (*Cluster 4*) species with increased abundance after both liraglutide and GUB09-145 treatment, and no differences between the two vehicle groups. *Left heatmap*: Spearman correlations between change in individual species abundance and various metabolic parameters affected by treatment, including plasma total cholesterol (TC), fasting glucose level on treatment day 14 (Fasting glucose), fasting terminal insulin levels (fasting insulin), glucose area-under the curve in an oral glucose tolerance test on treatment day 27 (AUC glucose OGTT), terminal plasma total triglycerides (TG), endpoint body weight loss relative to baseline (Body weight loss), and total energy intake during the treatment period (Total caloric intake). Asterisks denote significant correlation (FDR-corrected, p < 0.05) between individual species and relevant metabolic parameter. *Right heatmap*: Fold change (log_2_ transformed) in species abundance in individual mice as compared to baseline.

A total of 37 species significantly changed abundance in response to either liraglutide or GUB09-145 treatment (LMM, Treatment:Time, p < 0.05; post-hoc Tukey test, p < 0.05). The 37 species were grouped into four distinct clusters (Fig. 5, right panel). Cluster 1 comprised species with reduced abundance after liraglutide or GUB09-145 treatment; Cluster 2 comprised species with increased abundance primarily after liraglutide treatment; Cluster 3) comprised species with reduced abundance primarily after liraglutide treatment; and Cluster 4) comprised species with increased abundance after both liraglutide and GUB09-145 treatment. To assess the relationship between these regulated species and corresponding changes in the metabolic phenotype, within-subject change in the relative abundance of each individual species was correlated to six metabolic parameters assessed in the study (Fig. 5, left panel). Cluster 1 (15 species) was dominated by *Lachnospiraceae* and *Clostridiales* species and correlated to improvements in TC and glucose tolerance. Cluster 2 (5 species) comprised *Clostridiales spp*., *Burkholderiales bacterium YL45*, *Oscillospiraceae sp*. *and Akkermansia muciniphila*, which correlated to reduced body weight and caloric intake following liraglutide and GUB09-145 treatment. Cluster 3 (12 species) comprised *Clostridia* and *Clostridiales spp*., *Oscillospiraceae sp*., *Erysipelatoclostridium sp*., *Anaerotruncus sp*. *G3*(*2012*), *Bacteroidales sp*. *and Firmicutes* species that were associated with TC and reduced body weight. Cluster 4 (5 species) comprised *Enterococcus faecium* as well as *Clostridiaceae sp*., *Clostridium sp*. *and Desulfovibrionales* sp. and correlated to weight loss and reduced caloric intake.

### Functional implications of altered gut microbiome signatures

**Figure 6:**
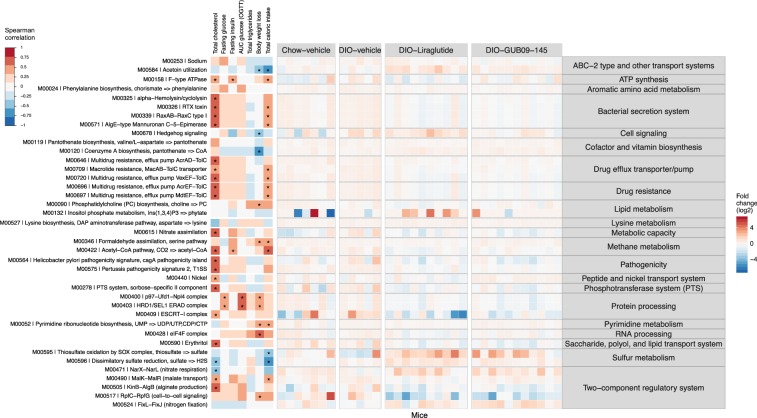
Bacterial KEGG metabolic pathways affected by treatment with liraglutide and GUB09-145. Heatmap depicting bacterial KEGG modules with changes over the course of liraglutide and GUB09-145 treatment. *Left heatmap*: Spearman correlations between change in individual species abundance and various metabolic parameters affected by treatment, including plasma total cholesterol (TC), fasting glucose level on treatment day 14 (Fasting glucose), fasting terminal insulin levels (fasting insulin), glucose area-under the curve in an oral glucose tolerance test on treatment day 27 (AUC glucose OGTT), terminal plasma total triglycerides (TG), endpoint body weight loss relative to baseline (Body weight loss), and total caloric intake during the treatment period (Total caloric intake). Asterisks denote significant correlation (FDR-corrected, p < 0.05) between species and relevant metabolic parameter. *Right heatmap*: Fold change (log_2_ transformed) in species abundance in individual mice as compared to baseline.

To evaluate microbial metabolic pathways associated with the gut microbiome compositional changes, the mouse gut microbiome genes were annotated with KEGG (Kyoto Encyclopedia of Genes and Genomes). Annotated categories included 603 KEGG common modules (found in ≥10% of the samples). Of these, 39 KEGG modules changed significantly after liraglutide or GUB09-145 treatment (LMM, treatment:time, *p* < 0.05, post-hoc Tukey test; p < 0.05). Figure 6 depicts abundance-shifted KEGG modules and associated functional pathways. Most conspicuously regulated pathways included Lipid metabolism, Protein processing, Sulfur metabolism, and Two-component regulatory system (phosphokinase-associated signal transduction). Whereas DIO control mice showed most consistent changes in KEGG modules associated with reduced bacterial sulfur metabolism, liraglutide and GUB09-145 effects on the gut microbiome were largely associated to effects on bacterial lipid metabolism (increased by liraglutide), sulfur metabolism (increased by both compounds), and Two-component regulatory system (modulated by both compounds).

## Discussion

Host-gut microbiota interaction is suggested to play a contributory role in the therapeutic effects of antidiabetics and weight loss promoting drugs. Among the various mechanisms proposed, the gut microbiota has been reported to modulate the expression and secretion of glucagon-like peptide hormones from enteroendocrine cells^27,61^. As GLP-1 and GLP-2 have become important targets in the treatment of type 2 diabetes, obesity and malabsorptive conditions^16,17,62^, we therefore characterized gut microbiome signatures in DIO mice treated with liraglutide, a selective GLP-1 receptor agonist, or GUB09-145, a potent dual GLP-1/GLP-2 agonist^26^.

16S rDNA amplicon sequence analysis and deep-sequenced shotgun metagenomics were applied for comprehensive mapping of gut microbiome changes caused by pharmacological intervention in DIO mice. Notably, gut microbiome signatures were highly homogenous within all individual experimental groups, which may be ascribed to the application of paired fecal samples to reduce interindividual variability in combination with a stable phenotype. This permitted detection of discrete, however consistent, alterations in the gut microbiome composition in DIO mice during the course of treatment. Compared to chow feeding, high-fat feeding for 35 weeks resulted in substantial rearrangement of the gut microbiota reflected by reduced bacterial community richness and diversity accompanied by highly consistent changes in the abundance of major bacterial divisions, dominated by phylum-level taxonomic shifts in *Firmicutes*, *Bacteroidetes*, *Proteobacteria* and *Actinobacteria*. This general gut microbial profile, including a higher *Firmicutes*:*Bacteroidetes* ratio, is in agreement with previous reports in high-fat diet fed mice^9,63,64^ and obese humans^5,6,65,66^. Notably, this characteristic microbial profile is reversible by dietary intervention^7,65,67^. *Firmicutes* are major producers of short-chain fatty acids (SCFAs), including butyrate, and this phylum has been proposed to play a contributory role in host adiposity by facilitating more efficient nutrient processing and energy extraction^6,9^. However, it should be noted that the biological relevance of the *Firmicutes/Bacteroidetes* ratio in predicting obesity has been disputed^8,68^. Also, as obesity-resistant mice assume an increased *Firmicutes*:*Bacteroidetes* ratio when fed a high-fat diet^63^, this emphasizes the importance of the diet as a major factor for gut bacterial community-wide changes in obesity.

Compared to the extensive gut microbiome alterations promoted by high-fat feeding *per se*, long-term liraglutide and GUB09-145 treatment led to relatively minor shifts in bacterial communities in DIO mice. Bacterial diversity measured at the species level did not change significantly following liraglutide and GUB09-145 treatment, which indicates that drug treatment effects on gut microbiome signatures in DIO mice were attributed to re-arrangement of low-abundant species. Liraglutide and GUB09-145 treated DIO mice showed notable commonalities in gut microbiome changes, dominated by reduced abundancy of species within *Firmicutes* (*Lachnospiraceae*, *Clostridiales*) and parallel increases in the abundance of species representing *Proteobacteria* (e.g. *Burkholderiales bacterium YL45*) and *Verrucomicrobia* (e.g. *Akkermansia muciniphila*) as well as *Firmicutes* (*Clostridiales*, *Oscillospiraceae*). The complex changes in gut bacterial distribution following liraglutide and GUB09-145 treatment is further underscored by drug treatment effects on bacterial species not regulated in DIO mice *per se*, including increased abundance of *Firmicutes* (*Clostridium*) and *Proteobacteria* (*Desulfovibrionaceae*) members.

Liraglutide has recently been reported to induce gut microbial structural changes in DIO mice, dominated by changes in the distribution of *Proteobacteria* and *Verrucomicrobia* phylotypes without affecting the abundance of *Firmicutes*^69^. Our study indicates that the largely unchanged proportion of *Firmicutes* following liraglutide treatment is explained by bidirectional changes in the abundance of bacterial species within this phylum. The complicated structural changes in the gut microbiome changes following liraglutide and GUB09-145 treatment therefore prompted us to perform correlations between changes in individual metabolic parameters and species abundance. Notably, body weight loss was correlated to increased abundance of *Akkermansia muciniphila*, a mucin-degrading SCFA-producing species which has been reported decreased in obesity and show negative correlation to markers of gut permeability and inflammation^70^. Although there is limited direct evidence for this species having functional effects on host metabolic profile, studies in DIO mice have demonstrated reduced adiposity and improved glucose tolerance following oral administration of *Akkermansia muciniphila*^71,72^. Importantly, both liraglutide and GUB09-145 have been reported to stimulate intestinal growth by increasing intestinal volume and mucosal surface area in mice^20,26^. It is therefore tempting to speculate if the intestinotrophic effects of GLP-1 and GLP-2 receptor agonists, leading to increased nutrient absorptive area, could stimulate colonization of mucosa-associated microbial communities. This hypothesis could be tested by assessing the growth properties of the relevant species or by doing histology on intestinal samples with probes directed at the differentially abundant species^73^. Another factor to be considered is the anorectic effects of liraglutide and GUB09-145^26,74^. As GLP-1 and dual GLP-1/2 receptor agonists have potent inhibitory effects on gastric emptying^26,74^, and correlations to increased abundance of species members of *Clostridiales*, *Burkholderiales*, *Oscillospiraceae* and *Akkermansia muciniphila* were driven by suppressed food intake in liraglutide and GUB09-145 treated DIO mice, this suggests that altered gastrointestinal nutrient flow could have influenced the abundance of these bacterial communities. Interestingly, the cholesterol-lowering properties of liraglutide and GUB09-145 were consistently correlated to reduced abundance of several members of *Firmicutes* (*Lachnospiraceae* and *Clostridiales spp*.). Intestinal cholesterol and cholesterol-derived primary bile acids can be metabolized by the colonic bacteria. In contrast to bile acid converting bacteria, only a few cholesterol-reducing bacteria have been isolated^75^. Several bacterial fermentation pathways have been proposed to lower cholesterol levels, including bile acid-converting and SCFA-producing enzymes which may reduce the rate of cholesterol reabsorption and increase cholesterol metabolism^76^. Whether these mechanisms are influenced by reduced abundance of *Firmicutes* must await further studies. It should also be taken into account that DIO mice show changes in the abundance of *Lachnospiraceae* depending on the dietary fat source^77^, and suppressed high-fat diet intake by liraglutide and GUB09-145 treatment could therefore potentially have impacted the composition of this bacterial family.

Compared to changes in species abundance, more discrete effects were observed on associated KEGG modules, which is likely explained by redundancy of functional metabolic features among regulated bacterial species. The effects of liraglutide and GUB09-145 were predominantly associated to increased bacterial lipid handling and sulfur metabolism. Interestingly, the drug effects on the thiosulfate oxidation module were unrelated to the metabolic phenotype, as vehicle-dosed DIO mice did not show any changes in these KEGG domains compared to chow-fed controls. In particular, correlations to sulfur metabolism were driven by increased abundance of *Desulfovibrionales sp*., an order mostly consisting of sulfate-reducing species. It is therefore possible that enhanced GLP-1/GLP-2 receptor activity facilitates production of substrates for production of gut bacterial hydrogen sulfide, which have anti-inflammatory properties and may enhance intestinal barrier function^78^.

Limitations of the study should be considered. No internal controls (*e*.*g*. extraction blanks) were applied to exclude/define potential contaminating microbial DNA originating from the extraction kit and other reagents used for sample DNA processing. As any potential background signal from the DNA extraction kit is expected to be substantially lower than the mock yield from the positive control (bacterial culture) and mouse fecal samples contained considerably higher bacterial DNA amounts compared to the positive control, this argues for the fecal samples almost solely consisting of biological relevant DNA. The study did not address effects of a selective GLP-2 receptor agonist, which makes it presently unresolved to what extent stimulated GLP-2 receptor function may have contributed to the gut microbiome changes following GUB09-145 treatment. Because the study did not include corresponding drug treatment in chow-fed mice, potential high-fat diet-dependent gut microbial compositional effects of liraglutide/GUB09-145 treatment cannot be ruled out. Also, it remains to be investigated whether the marked weight loss subsequent to suppressed caloric intake could be a major factor for shaping the gut microbiome in liraglutide/GUB09-145 treated DIO mice. This is relevant as weight loss following caloric restriction has been reported to influence gut microbiome composition in DIO mice, although the implications are poorly understood^33,79^. However, it should be emphasized that use of caloric restriction to control for weight-dependent effects in DIO mouse studies may potentially introduce confounding effects on microbiome signatures due to significant compensatory changes in hunger sensation, feeding pattern, resting metabolic rate and gut morphology which may be disproportionately to that attained by appetite suppressants^80,81^.

In conclusion, DIO mice assumed a phylogenetically similar gut microbiota composition following liraglutide and GUB09-145 treatment, suggesting that GLP-2 receptor stimulation played a marginal role in the microbiome modulatory effects of GUB09-145. The highly similar pattern of microbiome alterations following GLP-1 and dual GLP-1/GLP-2 receptor agonist treatment likely associate to the biological actions of the receptors that converge at several levels with respect to regulation of energy homeostasis, gut morphology and intestinal repair^82^. Whether the discrete shifts in gut bacterial species in DIO mice could potentially contribute to the metabolic effects of pharmacologically stimulated GLP-1 and GLP-2 receptor function or, alternatively, reflect gut microbiota adaptive responses secondary to reduced caloric intake must await further studies.

## Data Availability

All data generated or analyzed during this study are included in this published article.
